# Improving the Efficacy of Cognitive Training for Digital Mental Health Interventions Through Avatar Customization: Crowdsourced Quasi-Experimental Study

**DOI:** 10.2196/10133

**Published:** 2019-01-08

**Authors:** Max Valentin Birk, Regan Lee Mandryk

**Affiliations:** 1 University of Saskatchewan Department of Computer Science Saskatoon, SK Canada

**Keywords:** cognitive therapy, computer-assisted therapy, video games, attentional bias, cognitive bias, motivation

## Abstract

**Background:**

The success of internet-based mental health interventions in practice—that is, in the wild—depends on the uptake and retention of the application and the user’s focused attention in the moment of use. Incorporating game-based motivational design into digital interventions delivered in the wild has been shown to increase uptake and retention in internet-based training; however, there are outstanding questions about the potential of game-based motivational strategies to increase engagement with a task in the moment of use and the effect on intervention efficacy.

**Objective:**

Designers of internet-based interventions need to know whether game-based motivational design strategies can increase in-the-moment engagement and thus improve digital interventions. The aim of this study was to investigate the effects of 1 motivational design strategy (avatar customization) in an example mental health intervention (computerized cognitive training for attention bias modification).

**Methods:**

We assigned 317 participants to either a customized avatar or an assigned avatar condition. After measuring state anxiety (State-Trait Anxiety Inventory), we randomly assigned half of the participants in each condition to either an attentional retraining condition (Attention Bias Modification Training) or a control condition. After training, participants were exposed to a negative mood induction using images with strong negative valance (International Affective Picture System), after which we measured state anxiety again.

**Results:**

Avatar customization decreased posttraining state anxiety when controlling for baseline state anxiety for those in the attentional retraining condition; however, those who did not train experienced decreased resilience to the negative mood induction (*F*_1,252_=6.86, *P*=.009, *η*_*p*_^2^=.027). This interaction effect suggests that customization increased task engagement with the intervention in the moment of use. Avatar customization also increased avatar identification (*F*_5,252_=12.46, *P*<.001, *R*^2^=.23), regardless of condition (*F*_1,252_=.79, *P*=.38). Avatar identification reduced anxiety after the negative mood induction for participants who underwent training but increased poststimulus anxiety for participants who did not undergo training, further suggesting that customization increases engagement in the task (*F*_1,252_=6.19, *P*=.01). The beneficial effect of avatar customization on training was driven by participants who were low in their basic satisfaction of relatedness (*F*_10,248_=18.5, *P*<.001, *R*^2^=.43), which is important because these are the participants who are most likely in need of digital interventions for mental health.

**Conclusions:**

Our results suggest that applying motivational design—specifically avatar customization—is a viable strategy to increase engagement and subsequently training efficacy in a computerized cognitive task.

## Introduction

### Background

Internet-based mental health interventions are necessary to address a growing gap between an increased demand for treatment of mental health issues and the capacity of traditional therapeutic approaches to meet this growing demand [1]. It has been argued that internet-based mental health interventions have some benefits over traditional approaches, especially related to the increased accessibility of treatment (eg, because of geographical constraints of living in remote areas with access to clinical treatment), the ability to scale (eg, to seamlessly address the growing demand of people who could benefit from treatment), the ease of access (eg, through deployment of smartphones or websites), and the broad appeal (eg, to treat subclinical populations who may not qualify for treatment in health care systems that are already stretched in meeting the needs of clinical populations) [1-3].

To be successful, an internet-based mental health intervention requires *good intervention design* as evidenced by clinical efficacy. There are several examples of internet-based mental health applications that have demonstrated treatment effects [4,5] in randomized controlled trials (RCTs) [6-8]. However, because these mental health interventions are delivered digitally and often outside a laboratory or clinical context, success is not only defined by efficacy in *research* but also defined by efficacy in *practice*, which depends on validated implementation models and a focus on external validity—that is, demonstrated success in the context of intended use [4,5,9]. Success in practice requires *uptake* (ie, people have to use the applications), *retention* (ie, people have to use the applications over a long enough term to experience benefits), and demonstrated treatment effects under conditions of *practice* (ie, the clinical efficacy must translate into less controlled environments with multiple competing demands for a patient’s attention and time). These elements of demonstrated success in practice have been sources of failure for internet-based mental health interventions that have been effective in RCTs but are compromised by low uptake (ie, recruitment challenges [10]), attention (ie, failure to effectively engage participants in the moment of intervention use [11]), and retention (ie, failure in adherence over the medium term [12]) when delivered *in the wild*. Researchers have recently promoted a need for demonstrated success of treatment efficacy in both research and practice [5,9], and the failure to close the research-practice gap has prompted suggestions that researchers still have a lot to learn about how to implement these types of interventions in the wild [5].

### Uptake, Attention, and Retention

Researchers have argued that improving the in the wild success of internet-based mental health interventions can be facilitated by improving the *user experience design* [5]*.* Internet-based applications built for the purposes of leisure or enjoyment also depend on user engagement—that is, good uptake, attention, and retention—because the microtransaction model of monetization for the companies that build these applications requires that end users chose the application (uptake), engage with it (attention), and continue to use it (retention). Various interaction design strategies have been employed by leisure application designers to improve user engagement, including gamification—that is, the use of game-based elements in nongame contexts [13], the inclusion of extrinsic rewards [14], or leveraging social pressure to engage [15]. On the basis of increasing a user’s motivation by increasing their enjoyment of and invested effort in engaging with an application, these types of interaction design strategies are part of a growing field of research on motivational design for interactive technologies that have also been recently applied to the context of digital intervention design.

For example, applying motivational design principles has been shown to foster engagement with a 12-week neurofeedback treatment for children with fetal alcohol spectrum disorder [16], to increase motivation to use a stroke rehabilitation program [17], and to improve enjoyment of a social physical activity intervention for children with cerebral palsy [18]. Furthermore, a series of studies employing avatar customization (a motivational design strategy built on increased autonomy and identification) was shown to increase enjoyment of and effort invested in a game for training [19], to increase the time spent (free choice) in a game [20], and to combat attrition in an internet-based daily breathing exercise deployed over 3 weeks [21].

### Engagement in the Moment of Use

Previous work has suggested that motivational design principles could be employed to help close the research-practice gap for the design of internet-based mental health interventions by improving user engagement with the application [2]; however, prior work that led to these ideas has measured success through 2 approaches: first, by focusing on subjective measures of motivation, such as increased enjoyment of the intervention or perception of effort invested in the task (eg, [19]), and second, through metrics that operationalize intervention usage statistics, such as the time spent in treatment or number of returning sessions (eg, [21]). However, there is a third approach to characterizing success of motivational design as applied to digital interventions that has been underserved, that is, *metrics that characterize increased engagement with the task in the moment of use*. As researchers, we must differentiate between motivational design that results in greater exposure to treatment (ie, more time spent in training, more adherence)—which should result in improved efficacy through mere exposure and motivational design that fosters task engagement in the moment (eg, greater attention and focus and reduced response to distraction)—which should improve treatment efficacy without an accompanying increase in exposure.

In this paper, we employ 1 motivational design strategy (avatar customization) in an example mental health intervention (computerized cognitive training, CCT) to demonstrate that motivational design principles can not only improve exposure to treatment through greater uptake and retention but that they can improve focused engagement in the moment of intervention use.

### Computerized Cognitive Training as a Mental Health Intervention

CCT is one approach to intervention design with a focus on improving specific aspects of cognition. Feasibility studies on CCT have been shown to improve memory, self-control, reasoning, attention bias, and processing speed [22]; CCT has successfully been applied in clinical research to combat mental illness and cognitive deficits in disorders such as dementia [23], depression [24-26], neurodegenerative diseases [26], attention-deficit/hyperactivity disorder (ADHD) [27], and brain injury [28]. The most common CCT tasks are the Go/No-Go task [29], memory training [30], and the Attention Bias Modification Training (ABMT) task [31]. For example, Go/No-Go tasks require participants to inhibit responses under changing conditions (eg, in a fast-paced task, press L when a red box appears, but inhibit pressing L when a green box appears). Go/No-Go paradigms have been applied to the training of executive function [32], and research suggests that the paradigm can improve hyperactivity symptoms for children with ADHD [33] and reduce undesirable food intake when applied in the context of eating behavior [34].

Another approach to cognitive retraining is through attention bias modification [35], which exposes participants simultaneously to negative and neutral stimuli but reinforces an attentional shift toward neutral stimuli by presenting target probes behind only the neutral stimuli. ABMT has been shown to be an effective technique to shift a participant’s attention away from negative stimuli, to decrease self-reported anxiety, and to decrease the response to negative stimuli [31,36,37]. Although lab-based ABMT training has been shown to be effective [36], internet-based ABMT has generally not been shown to be effective, suggesting that the training task itself might require adjustment before dissemination over the internet [38]. In the case of training tasks that require the full attention of the patient, the lack of control over distractions and attention in the environment present in internet-based interventions may compromise treatment efficacy when delivered in the wild: for CCT to be fully effective, participants need to be vigilant, psychologically present, and engaged in the task.

CCTs have shown moderate-to-large effect sizes for improving attention, working memory, and global functioning [24]. However, to show effects in the wild, CCTs need to be designed in ways that maximize user engagement in the moment. Being inattentive, unfocused, or distracted will diminish the efficacy of attention-based training [39,40]. When CCT is applied in studies or during a session with a therapist, participants are externally regulated to focus on the task; however, this external regulation is drastically lessened when people engage in cognitive training during a commute, at home, or while they have a few minutes waiting in line. To support the success of internet-based mental health interventions in the wild, researchers need to ask, *how can we increase in-the-moment engagement to compensate for inattentiveness and distraction in the wild and subsequently improve training efficacy?*

### Engagement

Although internet-based mental health applications have increased the accessibility of treatment, their use still requires participant engagement and effort [11]. Theories of motivation provide guidance on how to design applications to maximize engagement. Self-Determination Theory (SDT), a well- established theory of human motivation [41], postulates that competence, relatedness, and autonomy are 3 predictors of motivation, which is expressed in terms of enjoyment, engagement, and effort. Competence—experiencing mastery over a task, autonomy—volitionally engaging in a task, and relatedness— experiencing connectedness to others—predict engagement and have been shown to be positively related to treatment outcome [11]. For example, clients who engage in therapy out of personal choice, that is, autonomy, are more likely to benefit from therapy. Increased volitional engagement has been shown to increase adherence and treatment efficacy [42].

#### Designing for Engagement

Multiple strategies have been applied to foster volitional engagement in a digital context. For example, gamification—the application of game elements in a nongame context [13]—has successfully been applied to increase volitional engagement in a variety of contexts [43]. Game-based training has been shown to improve working memory capacity [44], task switching ability (Ibid), visual short-term memory (Ibid), verbal reasoning (Ibid; [45]), visuospatial reasoning [46,47], response selection [48], visual attention [49,50], reaction time [51,52], and choice reaction time [53,54].

In a similar vein, persuasive technology uses strategies to bring about change by shaping or reinforcing behaviors or attitudes [55]. Two strategies commonly employed in persuasive technologies are personalization [56,57]—which is system-initiated tailoring that offers content or services personalized to an individual—and customization [56,58]— which is a system that supports user-initiated tailoring of content or services. Sundar et al [59] argue that although personalization will increase the relevance of content for individuals using an interactive system, customization yields systems and content that are not only relevant but also boost the agency and self-determination of the individual because it is they themselves who perform the tailoring. Personalization and customization have both been discussed as techniques to increase long-term engagement with internet-based interventions for mental health [21,60].

Customization fosters autonomy, a sense of control, and a sense of identity, making the person feel relevant in the context of their interaction [59]. As previously described, a series of studies employing avatar customization (which facilitated avatar identification) showed increased enjoyment of and effort invested in a game for training [19], increased the time spent (free choice) in a game [20], and combatted attrition in an internet-based daily breathing exercise deployed over 3 weeks [21]. Together, these studies show improvements in subjectively measured motivation and objectively measured motivation in terms of the time spent engaged with the application and number of return sessions in a 3-week intervention; however, there was no attempt to demonstrate improved treatment efficacy as a result of the increased engagement with the application in the moment of use. There was no differentiation between how the motivational design resulted in greater exposure to treatment and how the motivational design fostered task engagement in the moment (eg, more effort invested, better attention and focus, or reduced response to distractions), which should improve treatment efficacy without an accompanying increase in exposure.

Current digital mental health interventions require the attention and motivation of patients [5], which are unfortunately also characteristics that are in short supply for people who suffer from depression and could benefit most from treatment (Ibid). Previous work showed that avatar customization can increase motivation to engage with a training application and thus increase the time spent in training. In this paper, we suggest that employing motivational design principles—specifically the use of avatar customization—can increase the efficacy of a Web-based ABMT task by increasing task engagement *in the moment of training*, without requiring additional exposure to the treatment.

## Methods

### Research Questions

**Figure 1:**
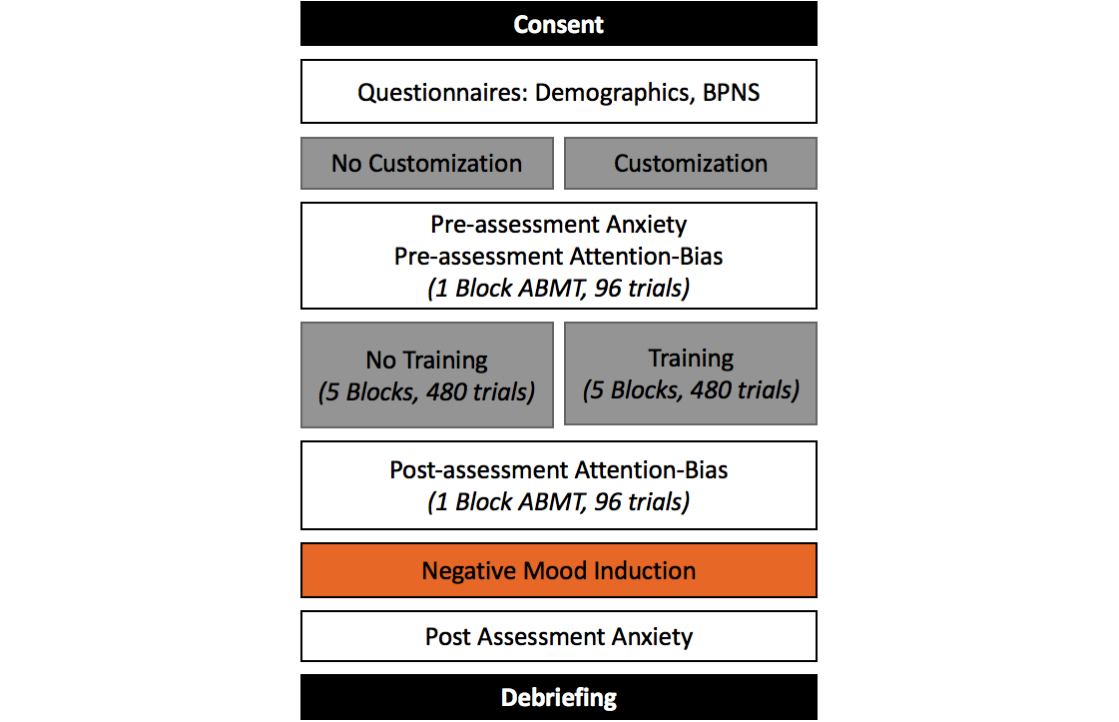
Experimental flow from consent (top) to debriefing. The experimental conditions are highlighted in grey. The negative mood induction is highlighted in orange. BPNS: Basic Psychological Need Satisfaction; ABMT: Attention Bias Modification Training.

We conducted an online study in which we asked half of the participants to customize an avatar; the other half were assigned a generic avatar. Each avatar group performed an ABMT task—half of the participants in each group were trained to preferentially attend to neutral over negative stimuli, the other half were not. Trained participants should be more resilient to subsequent negative stimuli; thus, participants were all subjected to a negative mood induction (viewing gruesome images) after completing the ABMT task and completed state anxiety scales before training and following the stimulus. Our experiment was a 2 (avatar: customized, generic) by 2 (attentional training, no training) between-subjects design; see Figure 1. Being inattentive, unfocused, or distracted will diminish the efficacy of attention-based training; thus, we ask whether the increased in-the-moment engagement as a result of avatar customization can improve training efficacy.

The following research questions (RQs) guided our analyses:

RQ1: Does customization improve the efficacy of attentional retraining?

RQ2: Does customization increase identification for trained and nontrained participants?

RQ3: Does avatar identification increase the efficacy of attentional training?

RQ4: Does training efficacy vary depending on basic needs satisfaction?

### Customization Using an Avatar Creator

**Figure 2:**
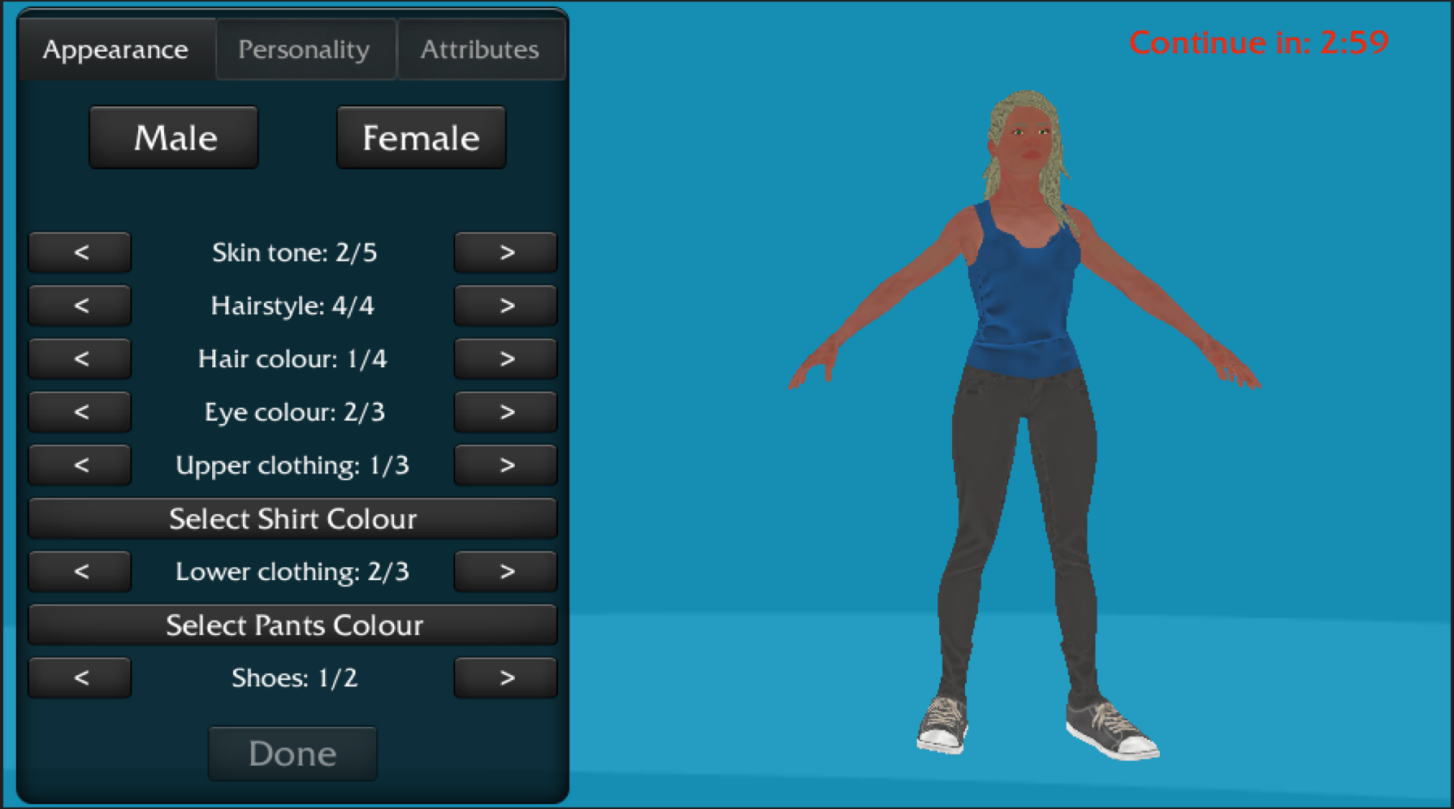
Picture of the avatar creator displaying the customization tool box on the left and a female avatar on the right.

To introduce customization, we used an avatar creator that has been shown to facilitate intrinsic motivation and invested effort in a game [20]. Participants were asked to create an avatar, choose its gender, and adjust its appearance, personality, and attributes (characteristics) in the same manner as described in the study by Birk et al [20]; see Figure 2. A minimum of 4 min in the character creator were required, but participants could take longer if they wished. After customizing their avatar, participants were shown a summary of their character.

**Figure 3:**
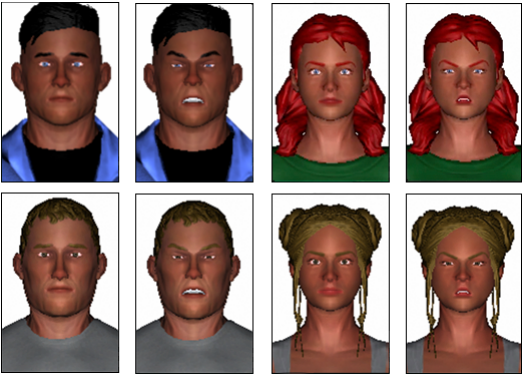
Customized (top) and generic (bottom) male and female avatars with neutral and angry facial expressions.

We presented half of our participants with the avatar creator; the other half were assigned an avatar that had generic features and medium hair and skin color (see Figure 3). Participants were asked their gender in a demographics survey; those who answered male or female were assigned an avatar of the same gender. Those who answered *other* (n=3) were then asked to choose a gender for their digital representation. For both generic and customized avatars, we created and stored 2 facial expressions: 1 neutral face and 1 angry face (see Figure 3). The faces were created by adjusting the 3D model of the face using an algorithm based on Ekman facial action coding system [61]. Due to the differences in facial geometry between male and female faces, there were 2 algorithms (1 for male, 1 for female), and all avatars of the same gender had the same algorithm applied. Once the avatars were customized or assigned, participants completed the ABMT task.

### Attention Bias Modification Training Task

**Figure 4:**
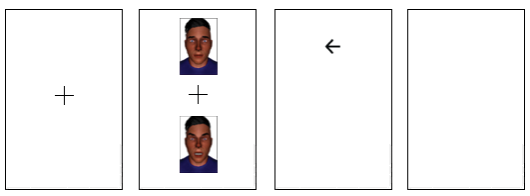
Attention Bias Modification Training trials. From left to right: fixation cross (500 ms), neutral/angry face (500 ms), probe (participants response), and intertrial interval (500 ms).

In each trial of the ABMT task [31], a fixation cross was presented for 500 ms centered in the screen. Following the presentation of the fixation cross, 2 avatar faces were displayed: 1 with a neutral facial expression and 1 with an angry expression. One face was displayed above the cross and 1 was displayed below the cross. After the faces were presented for 500 ms, they disappeared, and a probe was displayed behind one of the faces. The probe indicated either a right arrow or left arrow, and participants were asked to press the corresponding arrow key on the keyboard as quickly as possible. After pressing the left or right arrow key, an intertrial interval, showing a white screen, was displayed for 500 ms. The next trial started immediately with the presentation of the fixation cross. See Figure 4.

#### Attention Bias Modification Training: Training/No Training

Before beginning the ABMT task, participants were guided through a tutorial of 10 trials of neutral faces to learn the mechanics of the task. Participants were prompted to focus on the fixation cross and could only proceed if they pressed the correct arrow key as indicated by the probe.

Following the tutorial, the ABMT task was presented in 7 blocks: 1 preassessment block, 5 consecutive training/no training blocks, and a postassessment block. During each block, participants completed 96 trials (672 total). After each block, participants had a 6-second break, while being notified that the next block was about to start. This was done to indicate progress through the task.

We used 2 image sets of 4 avatar faces (2 male, 2 female) for the pre- and postassessment blocks; the order of the presentation of the 2 image sets was fully counterbalanced to avoid order effects. The avatars were selected from user-generated avatars from a previous study [20]. The presentation of sex (male, female), position (top, bottom), probe location (angry, neutral), and probe direction (left, right) was fully balanced over the 96 trials.

In the ABMT training condition, probes only appeared behind neutral faces, with the intent to shift participants’ attention toward neutral stimuli. In the no training condition, participants were exposed to a fully balanced probe presentation similar to the assessment condition in which 50.0% (240/480) of the probes appear behind the neutral faces and 50.0% (240/480) behind the angry faces. See the study by Hakamata et al [31] for a detailed description of the ABMT task.

After each of the 7 trail blocks, participants completed a mood scale with 7-point agreement to 4 states (I feel: relaxed, happy, depressed, anxious) representing the 4 corners of arousal-valence space [62].

#### Trial Removal and Logging

Incorrect responses to the probes were removed from subsequent analysis because they show that the participant did not pay attention in the trial. Individual trials were also removed when participant’s response time was greater than 3 SDs of their own mean performance over both assessment blocks. We logged the position (top, bottom), gender (male, female), and expression (angry, neutral) of the probe location; the response time (in ms); and the correctness of the probe response (true, false).

### Negative Mood Induction

To measures resilience to a negative mood induction, we presented 20 negative images from the International Affective Picture System (IAPS; [63])—ID: 2703, 3010, 3015, 3225, 3230, 3350, 3530, 3550.1, 9040, 9265, 9301, 9410, 9420, 9433, 9490, 9500, 9570, 9611, 9635.1, and 9901. Images were selected based on valence (mean=2.01, SD=0.5, min=1.51, max=3.60) and arousal (mean=5.92, SD=0.63, min=4.34, max=7.16). To ensure that participants looked at the images, we asked them to rate each image using the valence and arousal scales of the visual self-assessment manikin [64]—valence and arousal scales were sequential to increase the time spent looking at each image. IAPS images have previously been used as a negative mood induction in the context of the ABMT [31]. Descriptively, participants show a similar response to the negative images as with normative IAPS ratings (valance: mean=1.91, SD=0.62, min=1, max=4.05; arousal: mean=4.97, SD=1.215, min=1, max=7), indicating that the images were perceived as expected.

### Participants and Deployment Platform

We recruited 317 participants through Amazon’s Mechanical Turk (MTurk). MTurk acts as a broker between parties offering a range of human intelligence tasks and paid workers. Although MTurk has been shown to be reliable as a recruitment tool for research [65-67], we excluded participants from analysis if they were not performing the task with care, which we determined in multiple steps. We removed 33 participants based on missing trials (indicating technical difficulties) or too many trials (indicating that they reloaded the task part way through). Then, we calculated variance within each survey subscale and removed participants (n=8) from subsequent analyses who demonstrated response variance greater than 3 SDs above mean variance on 3 or more questionnaire subscales. Having high variance within a subscale is indicative of not paying attention to the survey questions and the reverse-coded items. We also removed participants from subsequent analyses who completed 2 or more questionnaires with an average time below 1 SD of the average response time (n=10). Finally, we removed participants who spent more than 2 min answering the state anxiety questionnaire after the negative mood induction (n=4), as it would indicate that they were taking time to recover while answering the questions.

After the outlier participants were excluded, 262 participants remained. As we controlled our analysis for age and gender, we also excluded the 3 participants who identified their gender as *other*, leaving 259 participants (51.0% [132/259] female, mean age=35.3 years, SD=11.5) in all of our analyses. Participants received compensation of US $10 for their participation. Ethical approval was obtained from the University of Saskatchewan behavioral research ethics board, and participants were asked to give informed consent at the beginning of the task. To comply with ethical guidelines, the task was only available to workers who were older than 18 years. Additionally, only workers from the United States with an approval rate above 90% were offered the task as a means of quality control, and a trigger warning was provided at multiple points before the negative images being presented.

### Measures

Identification was measured using the avatar-related subscales of similarity identification, embodied identification, and wishful identification from the Player Identification Scale [68]. Participants rated their agreement to identification-related statements—“My character is like me in many ways”—on a 7-point Likert scale. Identification has been shown before to be an important construct factor when customizing avatars [20].

State anxiety was measured using the state scale from State-Trait Anxiety Inventory (STAI; [69]). Participants rated how well statements—for example, “I’m calm”—described their current state on a 4-point scale from “Not at all” to “Very much.” STAI has been successfully been used before in research on resilience to negative affect [70].

Basic needs satisfaction was measured using the Basic Psychological Need Satisfaction (BPNS; [71]) scale. BPNS scale includes subscales for the basic satisfaction of competence, autonomy, and relatedness, as 3 ongoing needs that when satisfied lead to optimal development and function. Participants rated their agreement to statements—“People I know tell me I am good at what I do”—on a 7-point Likert scale. The scale has been used before in research on resilience to negative affect [71].

### Procedures

Participants were informed about time (60 min) and payment (US $10), procedure, and the fact that the study included gruesome imagery. After giving consent, participants were asked to fill in questionnaires on their demographics, basic needs satisfaction, and the questionnaire for baseline state anxiety.

**Table 1:** Descriptive statistics for dependent and control variables displayed by avatar customization and training condition.

Avatar customization			ABMT^a^ training	
			No training	Training
**STAI^b^ (pre), mean (SD)**				
	No customization		1.95 (0.56)	2.05 (0.64)
	Customization		1.94 (0.58)	1.98 (0.57)
**STAI (post), mean (SD)**				
	No customization		2.37 (0.59)	2.6 (0.67)
	Customization		2.55 (0.58)	2.37 (0.59)
**Identification, mean (SD)**				
	No customization		2.63 (1.35)	2.62 (1.29)
	Customization		4.11 (1.33)	3.76 (1.27)
**Relatedness, mean (SD)**				
	No customization		4.97 (1.29)	4.97 (1.08)
	Customization		5.15 (1.02)	5.02 (1.13)
**Age (years), mean (SD)**				
	No customization		37.75 (10.83)	37.75 (10.83)
	Customization		32.2 (9.29)	32.2 (9.29)
**Gender, n (%)**				
	**Male**			
		No customization	33 (51.6)	27 (46.6)
		Customization	39 (54.9)	34 (51.5)
	**Female**			
		No customization	31 (48.4)	31 (53.4)
		Customization	32 (45.1)	32 (48.5)

^a^ABMT: Attention Bias Modification Training

^b^STAI: State-Trait Anxiety Inventory

Participants were then assigned to 1 of the 4 conditions: customized avatar/ABMT training, customized avatar/no ABMT training, generic avatar/ABMT training, and generic avatar/no ABMT training. For descriptive statistics, see Table 1.

Depending on the condition, participants either customized an avatar or were assigned a generic avatar and started immediately with the ABMT tutorial. After the tutorial, all participants did a block of preassessment trials with 1 of the previously described image sets. Following this initial block, half of the participants received 5 blocks of attentional retraining, with probes only behind neutral faces, whereas the other half completed 5 blocks, with the probe appearing equally behind neutral and angry faces. All participants performed 1 block of post assessment after training/no training with the other image set. Following assessment, all participants were exposed to the negative mood induction using the gruesome IAPS images. Following the negative mood induction, participants filled in the post state anxiety questionnaire. Finally, participants provided information about how much they identified with the avatar face that was used for training/no-training. Participants were then debriefed about the purpose of the experiment and were directed to adorable images of baby animals if they wanted to combat the negative mood induced by the IAPS images. See Figure 1 for the experimental flow.

### Data Collection and Analysis

All data were logged to a database on a server at the University of Saskatchewan and were analyzed using Statistical Package for the Social Sciences (SPSS) version 24 (IBM Corp) with the process macro for moderation and mediation analyses [72].

Before analyzing the effects of training and customization on anxiety, we analyzed the block-based mood ratings to ensure that the different conditions did not directly induce different vulnerability to the negative mood induction. There were no significant interactions with either avatar customization or training, implying that the conditions did not directly influence mood (see [73]).

We computed 4 models. First, we investigated the role of avatar customization and attentional retraining on state anxiety after the negative mood induction. We use an analysis of covariance (ANCOVA) with avatar (customized, generic) and attentional retraining (training, no training) as factors on the dependent measure of state anxiety measured after the negative mood induction.

The ANCOVA allowed us to control for levels of state anxiety before attentional retraining, age, and sex by including these variables as covariates. ANCOVAs have been shown to have higher power in randomized studies with a baseline compared with using repeated measures analysis of variances (ANOVAs) [74].

The next 3 regression models were all controlled for baseline state anxiety, age, and gender. In our second model, we considered whether avatar customization (X) predicts identification (Y) and whether training (M) moderates this relationship (model=1; [72]). The link between avatar customization and identification has been previously established [20,75].

Third, we considered whether identification (X) predicts anxiety (Y; post negative mood induction) and whether training (M) moderates this relationship (model=1; [72]).

Finally, we conducted a moderated moderation (model=3; [72]). We considered whether avatar customization (X) predicts anxiety post negative mood induction (Y), whether this relationship is moderated by baseline need satisfaction (M), and whether that moderation is moderated by training (W). A moderated moderation model is similar to a 3-way interaction in an ANOVA but allows for the inclusion of continuous variables as factors.

## Results

We created 4 statistical models, as described above, and used these models to answer a series of research questions.

### RQ1: Does Customization Improve the Efficacy of Attentional Retraining?

**Figure 5:**
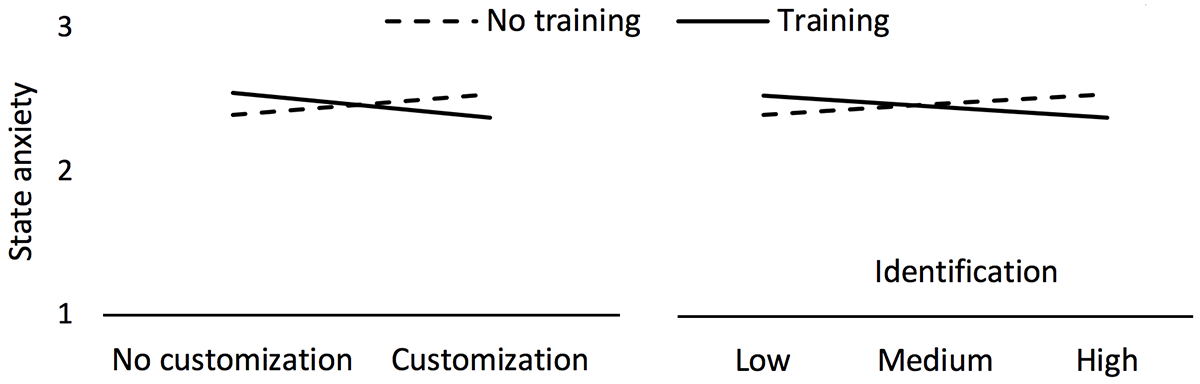
Left: state anxiety for the interaction of training and avatar customization (RQ1). Right: state anxiety for the interaction of training and identification (RQ3). Displayed data are controlled for age, gender, and baseline state anxiety.

Participants in the ABMT training group who customized their avatar should show increased resilience to negative stimuli. An ANCOVA controlling for pretraining anxiety, age, and gender showed a significant interaction between avatar customization and attentional retraining on anxiety measured after the negative mood induction (*F*_1,252_=6.86, *P*=.009*, η*_*p*_^2^=.027*)*. Bonferroni- corrected post hoc tests showed that training reduces state anxiety only when participants used a customized avatar (*P*=.04); when a generic avatar was used, training was not more effective than no training (*P*=.10). This result suggests that attentional retraining is more effective when participants were allowed to create a customized avatar and were presented with personalized stimuli (see Figure 5).

Although the interaction of avatar and training shows a significant effect, the partial eta-squared value (*η*_*p*_^2^=.027) implies a small effect. The large effect on poststimulus anxiety (*R*^2^=.401) is mostly explained by baseline anxiety (*η*_*p*_^2^=.353). However, when looking at the model by individual effect sizes of the included variables (age, gender, training, avatar, and the interaction of training and avatar), we find that age (*η*_*p*_^2^=.032) and gender (*η*_*p*_^2^=.030) both show small effects. Training and avatar (both not significant) explain almost no variance (*η*_*p*_^2^<.001 and *η*_*p*_^2^=.001, respectively). The interaction of avatar and training (*η*_*p*_^2^=.027) shows an effect similar in size to the effects of major demographic variables (ie, age and gender) on anxiety.

These results are in line with previous research, showing that customization increased identification with a digital representation, and as a result increased positive affect, task enjoyment, invested effort, and motivated behavior measured as time spent in a free choice game [20], and that these effects are of a similar size as the effects of major demographic variables.

### RQ2. Does Customization Increase Identification for Trained and Nontrained Participants?

**Figure 6:**
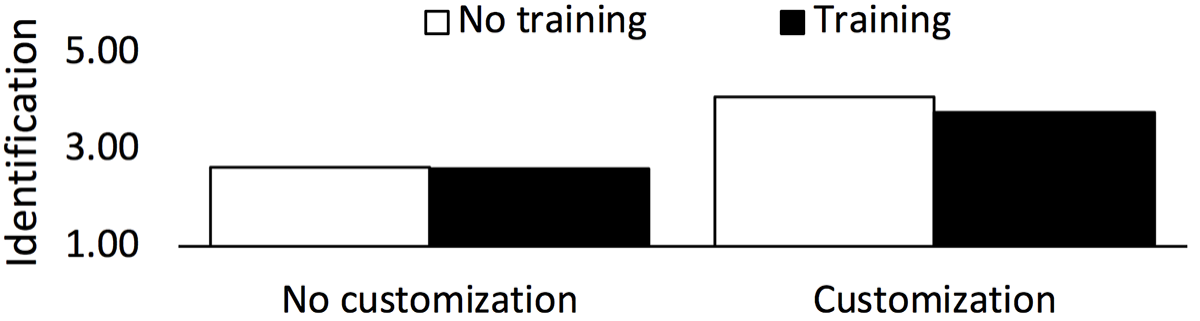
Effect of training and customization on identification (RQ2). Displayed data are controlled for age and gender.

We investigated the prediction of avatar customization on avatar identification, moderated by training. The model was significant (*F*_5,252_=12.46, *P*<.001, *R*^2^=.23). Customization predicts identification (beta=.651, *P*<.001); however, the nonsignificant interaction with training (*F*_1,252_=0.79*, P*=.38) shows that the prediction does not depend on whether the probe appeared under angry or both angry and neutral faces (see Figure 6).

### RQ3. Does Avatar Identification Increase the Efficacy of Attentional Training?

We investigated the prediction of avatar identification on anxiety measured after the negative mood induction, as moderated by training. The model was significant (*F*_6,252_=28.49, *P*<.001, *R*^2^=.40). There was neither a significant main effect for identification (*P*=.99) nor for training (*P*=.81). However, the interaction between identification and training (*F*_1,252_=6.19, *P*=.01) shows that for participants who underwent training, identification tends to reduce anxiety, that is, increases training efficacy; however, for participants who did not undergo training, the trend is in the other direction—greater identification increases anxiety (see Figure 5).

### RQ4. Does Training Efficacy Vary Depending on Basic Needs Satisfaction?

**Figure 7:**
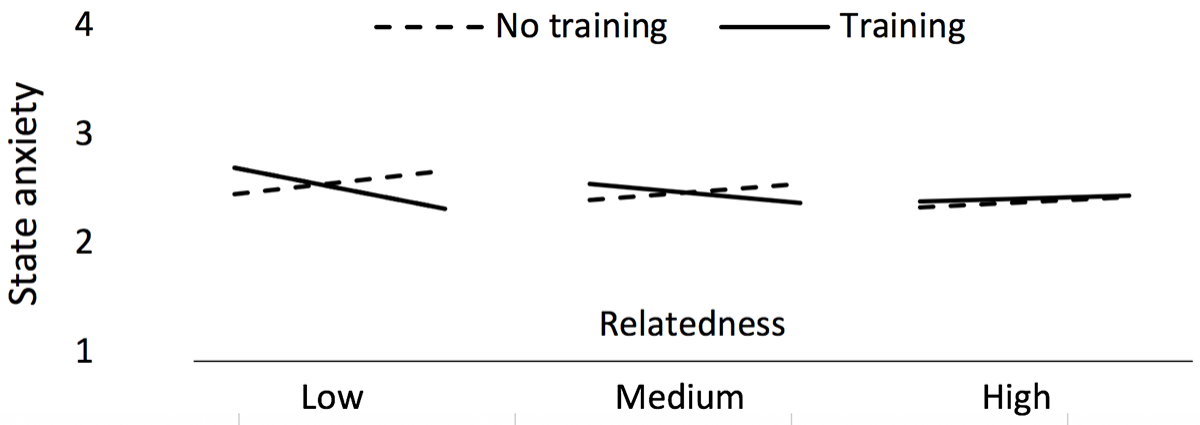
Moderation model showing avatar on state anxiety moderated by satisfaction of relatedness and then moderated by training (RQ4). Displayed data are controlled for age, gender, and baseline state anxiety.

We showed in R1 that avatar customization and training interact to yield lower anxiety. Now we ask whether this effect is being driven by participants with varying levels of basic psychological needs satisfaction. We used basic satisfaction of relatedness as it has been shown to be an important predictor of depression, addiction, and other mental disorders [76]. A moderation model with the predictor avatar (X) on state anxiety (Y) moderated by satisfaction of relatedness (M) and then moderated by training (W) was significant (*F*_10,248_=18.5, *P*<.001, *R*^2^=.43); we controlled for baseline state anxiety, gender, and age. As expected, the interaction between avatar and training was significant (*P*=.007). Most interestingly, the interaction between satisfaction of relatedness, avatar, and training also was significant (*P*=.02). This 3-way interaction showed that the interaction between avatar customization and training is more pronounced for those lower in satisfaction of relatedness (see Figure 7).

Participants with lower relatedness satisfaction were driving the significant interaction between avatar customization and training, which is a meaningful result, as these are the participants who are most likely to be in need of a mental health intervention in the first place [76].

## Discussion

We summarize our findings, contextualize them in theory, and discuss the implications, limitations, and opportunities for future work.

### Principal Findings

The results revealed 4 main findings:

1. First, avatar customization increased resilience to a negative mood induction for participants who trained attentional bias, compared with those who did not train attentional bias. Thus, avatar customization improved training efficacy, presumably as a result of increased in-the-moment engagement.
2. Second, avatar customization increased avatar identification, regardless of whether or not participants trained attentional bias.
3. Third, avatar identification tended to reduce anxiety after the negative mood induction for participants who underwent training but tended to increase poststimulus anxiety for participants who did not undergo training, suggesting that customization increases in-the-moment engagement.
4. Fourth, the beneficial effect of avatar customization on training is being driven by participants who are low in their basic satisfaction of relatedness, which is important because these are the participants who are most likely in need of digital interventions for mental health.

### Explanation of Findings

Our findings suggest that attentional retraining is improved when participants are more engaged and invested in the task. On the basis of prior research [20], we assume that increased motivation and willingness to invest effort increases attention to the task and efficacy as a result. We further explain potential mechanisms of why avatar customization increased treatment efficacy.

#### Self-Determined Experience

The results presented in this study and previous research using avatar customization [20] show that customization increases identification and that identification positively predicts task engagement. Engagement can be understood as an increase in participants’ invested effort, a central construct in describing intrinsic motivation and task engagement [77]. Research on motivation [78,79] suggests that invested effort depends on factors related to the experience itself, in particular, whether or not the interaction satisfies our needs for competence (ie, feeling a sense of mastery over challenges), autonomy (ie, experiencing the choice to engage under our own volition), and relatedness (ie, feeling connected to others) [80]. In our experiment, we aimed to increase motivation by manipulating choice as a means of increasing autonomy, that is, participants either customized an avatar or they were assigned a generic avatar. Exercising choice has motivational benefits, increasing the sense of volitional control and ownership over analog and digital objects [81,82].

SDT further suggests that intentionally engaging in a task is an important predictor of outcome— presumably because of the increased attention and focus paid to the task at hand. A more positive attitude toward the task—facilitated, for example, by feeling volitional control over the content or ownership over objects—may lower cognitive resistance to engagement. Participants in the customization condition may have been less likely to reject engaging with the task simply because it did not appeal to their preferences. The ABMT—like other CCT tasks—is not a particularly entertaining task, so frustration and boredom have the potential to undermine the positive effects of the training; however, we assume potential boredom and frustration are partially counteracted by the positive effects of customization.

#### Personalized Experience

We have argued that avatar customization increased efficacy by increasing autonomy and invested effort or by improving attitude and feelings of control. In addition, the customized avatar condition presents tailored stimuli, that is, customized avatar faces with neutral or negative expressions, during the training phase. Tailoring (through customization or personalization) is a persuasive strategy that has been used, for example, to increase motivation to play serious games [83] and to improve the efficacy of serious games for changing attitudes, intentions, and self-efficacy around healthy eating behavior [84]. The personalization of the stimuli in our experiment may have directly affected participants’ perception of the stimuli, making the stimuli’s emotions (neutral, negative) more salient to them.

#### Individual Differences

Our results suggest that the customized avatar increases engagement in the moment of use and subsequently training efficacy; however, we also show that the improvements resulting from avatar customization were most pronounced for those participants who experience low satisfaction of relatedness, that is, people who feel less connected to others, experience less support, or even feel lonely. Our intervention facilitates choice but enhances the experience of relatedness in the application. Although we did not explicitly measure relatedness satisfaction during the task, prior research has shown that customization can facilitate feelings of connectedness with a digital representation [80]. Increased satisfaction of relatedness might have improved engagement with the training task involving an avatar. This is important because loneliness, feelings of social exclusion, and feeling disconnected from supportive social groups are predictors of vulnerability for many mental health conditions such as depression and anxiety [85].

#### Increased Engagement With No Training

Avatar customization increased resilience to negative stimuli after the attentional bias modification training (ie, saw probes appear only under neutral faces); however, we also observed that the group that used avatar customization without training (ie, saw probes appear under both angry and neutral faces) was actually *more* susceptible to the negative mood induction. These results suggest that avatar customization increased task engagement, independent of the training condition. For participants who trained, this resulted in better resilience to the negative stimuli than those who trained with a generic avatar face; however, for participants who did not train, this likely resulted in them investing more attention overall and this being more susceptible to the negative mood induction than those who did not train using the generic avatar faces. This interaction result is not surprising, considering prior evidence of the relationship between avatar customization, identification, and motivation [20] in increasing task engagement, and further supports our arguments for customization as a motivational design strategy that can increase in-the-moment engagement.

#### Blending and Extending Existing Therapeutic Approaches

There are a variety of existing approaches to maximize the efficacy of internet-based interventions in mental health. For example, blended interventions, that is, interventions that blend internet-based forms of therapy with the interleaved presence of a therapist, have been shown to decrease the load on therapists and are similar in effect to traditionally delivered cognitive-behavioral therapy (CBT) programs [86,87].

Stand-alone internet-based CBT programs [88,89] have also been evaluated in RCTs and have shown promise for the improvement of mental health and well-being. For example, modular internet-based CBT programs such as MoodGYM [6], SilverCloud [90], or the mobile-based suite IntelliCare [3] have been shown to successfully improve mental health in cases of depression or anxiety. However, limitations of internet-based CBT programs such as the reading and language requirements, financial and time costs of localization, and the required time investment that is difficult for users to achieve suggest that other approaches might need to be deployed in tandem. Furthermore, CCTs such as ABMT have been applied adjacent [91] to internet-based CBTs, showing potential in blending approaches.

Although our study only considers customization in the context of bias modification, there are other approaches to internet-based intervention design that may benefit from the increased task engagement that we demonstrate. For example, including customized avatars to guide a patient through a CBT application or including other personalized stimuli could potentially increase engagement in the moment or result in increased adherence, as suggested by Birk et al [21]. Or consider digital phobia treatments that expose patients to fear-inducing stimuli (eg, [92-94]), which could be even more effective if patients are able to customize the presented stimuli, thereby increasing salience in an individual patient’s personal context. And finally, consider narrative-based therapeutic applications for people who suffer from post-traumatic stress disorder, which walk a patient through their experiences and help them to reframe the traumatic event (eg, [95,96]). Supporting patients to personalize the narrative, graphical objects, and other intervention elements could increase the efficacy of this important type of mental health intervention.

Until we validate innovative methods, adapting well-evaluated approaches to be delivered at scale is a safe and promising way forward. Exposing people to techniques that are not ready for use as treatment has associated dangers, whereas investigating subtle adaptations to existing approaches—such as interface customization applied to existing treatments—may improve and optimize established interventions.

Interface customization is not an intervention in itself but rather an enhancement that can be applied across a range of existing interventions. From the perspective of implementation medicine—the branch of medicine that asks if a research result should be implemented in practice [97]—it is a requirement to have evidence-based proof that a technique is either more effective than prior techniques or at least provides equal effectiveness with a lower investment of time and/or money [98]. Our research shows that tweaks to existing interventions such as adding customization can significantly improve in-the-moment engagement.

#### Supporting Motivation in the Immediate and Long Term

In this research, we show how avatar customization can increase task engagement and focused attention in the moment of application use. Motivational theories—such as SDT—describe how fostered autonomy can increase the effort invested in a task, which we suggest results in subsequent task engagement during intervention use. In contrast, our previous work on avatar customization has shown increased adherence to a daily training regimen over a medium-term (3-week) breathing intervention [21]. It is important to distinguish the motivational benefits that accrue from increased exposure to treatment (ie, through increased log-ins or more persistent usage) and those that result from increased attention in the moment of intervention use, without any accompanying increase in treatment exposure. Customizing avatars has been shown to increase motivation both in terms of increased treatment exposure through retention [21] and—in this paper—through focused attention in the moment. Additionally, personalization has been explored as an approach to enhance long-term engagement in the context of digital interventions for mental health [60]. Further work is needed to explore the potential additive effects of these 2 approaches in increasing motivation and to explore the mediating motivational factors (eg, attention, enjoyment, and effort) that could explain the improved outcome observed in this study.

### Limitations and Future Work

Our study has limitations that we intend to address in future work.

First, identification occurs in multiple ways, that is, wishful, similarity, and embodied identification [99]; in this paper, we did not manipulate the different aspects of identification, which potentially could enhance or diminish the efficacy of customization. Using, for example, nonhumanoid representations in a customization procedure might differentially affect identification and efficacy of a subsequent task. We plan to investigate different types of customization to explore the differential effects of identification types on intervention efficacy and to determine whether customization of other interface elements (beyond faces) can have the same benefits.

Second, the experimental context needs to be considered for the interpretation of our results; participants were recruited using a crowdsourcing platform and were paid for their participation, which creates a different experience than being exposed to attentional retraining as part of a therapy for participants who are in need of treatment.

Third, the attention bias modification task is only 1 potential digital intervention. Enhancing efficacy through customization needs to be tested across various interventions. Enhanced engagement through customization will particularly benefit interventions that rely on focused attention, such as cognitive tasks like the ABMT. How customization can integrate with other therapeutic approaches (eg, CBT-based interventions) remains to be investigated. Our technique requires very little effort to implement but shows significant changes of efficacy. How to leverage volitional engagement to best increase the efficacy of a variety of interventions is a promising direction; however, more systematic research on the limits of customization for in-the-moment task engagement needs is required.

Fourth, our study does not distinguish whether the effects are a result of participants coping better with the negative mood induction post training or if the effects are a result of the training itself. The former would suggest that the participants developed better strategies to disconnect from maladaptive thought processes, whereas the latter suggests that training helps participants protectively shift attention away from negative cognitions.

Fifth, this study focuses on the effects of in-the-moment engagement. However, many tasks (including ABMT) require frequent repetitions, for example, being used for 30-min daily. Although previous work suggests that avatar customization has positive effects on long-term engagement [21], we did not specifically investigate the effects of avatar customization over the long term in this study.

### Conclusions

Mental illness is increasing, yet therapies have not adapted to meet the growing demand. Computerized interventions delivered at scale have the potential to ease clinical demand and interventions accessible for those who do not qualify for traditional therapies or cannot access or afford them. However, the efficacy of computerized training cannot be sacrificed in service of a wider reach. Our results suggest that increasing in-the-moment engagement through interface customization and personalization can increase training efficacy.

We asked participants to complete online ABMT with a customized avatar or an assigned generic avatar. ABMT helps people shift their attention away from negative stimuli and has been shown to increase affective resilience to a subsequent negative mood induction (eg, rating gruesome images). Our results showed that a version of the ABMT using customized interface elements generated through avatar customization increased resilience to a subsequent negative mood induction, suggesting that avatar customization increases in-the-moment engagement, and subsequently training efficacy. Furthermore, the customization benefits were particularly pronounced for participants with low satisfaction of relatedness, who are most at risk for developing mental illness.

Digital interventions delivered at scale offer a promise of increasing the reach of mental health treatment to a greater number of people in a wider range of places. Our work shows that avatar customization may help to improve the efficacy of existing and future training programs delivered in the wild.

## Abbreviations

ABMT: Attention Bias Modification Training
ADHD: attention-deficit/hyperactivity disorder
ANOVA: analysis of variance
ANCOVA: analysis of covariance
BPNS: Basic Psychological Need Satisfaction
CBT: cognitive-behavioral therapy
CCT: computerized cognitive training
IAPS: International Affective Picture System
MTurk: Amazon's Mechanical Turk
RCT: randomized controlled trial
RQ: research question
SDT: Self-Determination Theory
STAI: State-Trait Anxiety Inventory
